# Advice and care for patients who die by voluntarily stopping eating and drinking is not assisted suicide

**DOI:** 10.1186/s12916-017-0994-2

**Published:** 2017-12-27

**Authors:** Andrew McGee, Franklin G. Miller

**Affiliations:** 1 Australian Centre for Health Law Research, Faculty of Law, Queensland University of Technology, 2 George St, Brisbane, Qld 4001 Australia; 2 Medical Ethics in Medicine, Weill Cornell Medical College, 425 East 70th Street, New York, NY 10021 USA

**Keywords:** Physician assisted suicide, Right to refuse food and water, Voluntary palliated starvation, Voluntarily stopping eating and drinking, VSED

## Abstract

**Background:**

A competent patient has the right to refuse foods and fluids even if the patient will die. The exercise of this right, known as voluntarily stopping eating and drinking (VSED), is sometimes proposed as an alternative to physician assisted suicide. However, there is ethical and legal uncertainty about physician involvement in VSED. Are physicians advising of this option, or making patients comfortable while they undertake VSED, assisting suicide? This paper attempts to resolve this ethical and legal uncertainty.

**Discussion:**

The standard approach to resolving this conundrum has been to determine whether VSED itself is suicide. Those who claim that VSED is suicide invariably claim that physician involvement in VSED amounts to assisting suicide. Those who claim that VSED is not suicide claim that physician involvement in VSED does not amount to assisting suicide. We reject this standard approach.

**Conclusion:**

We instead argue that, even if VSED is classified as a kind of suicide, physician involvement in VSED is not a form of assisted suicide. Physician involvement in VSED does not therefore fall within legal provisions that prohibit VSED.

## Background

Cases of people with debilitating medical conditions who seek to die by voluntarily stopping eating and drinking (VSED) have been documented in the professional literature [1–8]. However, there remains a lack of clarity about how to characterize this option and its legal ramifications in jurisdictions where, although suicide itself is not unlawful, assisting suicide is a crime. Since suicide is not itself unlawful, the focus has been on whether a physician who provides care and palliative relief to a patient who is undertaking VSED is in effect assisting suicide. Many commentators and some instances of case law address this by taking the view, often dogmatically, that VSED does not constitute suicide [9, 10]. The logic behind this position is that, if VSED is not suicide, then it follows that physician involvement is not assisted suicide. If, by contrast, VSED is suicide, then it would be natural to presume that physician involvement is assisted suicide [2, 8].

In this paper, we reject the assumption that the ethical and legal status of physician involvement in VSED is tied to whether VSED is suicide. We instead contend that, while there are good reasons for claiming that VSED is a form of suicide, there are equally good reasons for claiming that physicians are not assisting suicide when offering palliative care for VSED or when providing advice about the availability of this option. The issue of whether palliative care for VSED is assisting suicide is the key issue since many patients and physicians do, or will, think that VSED is a form of suicide, and physicians need to know whether they are at risk of violating the law (in places where assisting suicide remains unlawful) by providing palliative care for those who choose to die by VSED. Through this discussion, we aim to explain why these two issues are logically separate. After briefly explaining the reasons for characterizing VSED as a form of suicide, we will examine the issue of assisted suicide.

## Discussion

### Is VSED suicide?

VSED is distinct from the common tendency of terminally ill patients to lose interest in eating near the end of life. VSED is a form of suicide because there is unquestionably an intention to bring about one’s own death. Unlike with life-extending treatment, where competent patients can argue that they merely intend to be free of burdensome treatment, with death a foreseen side-effect, in VSED no effect can meaningfully be said to be intended other than death [9]. Equally, the patient’s aim is to bring about death sooner than it might occur naturally in VSED; life is not being extended by life-prolonging measures such as mechanical ventilation, and so it is not possible to argue that the patient is merely allowing themselves to die from a condition which would already have killed them without such measures. Therefore, VSED represents an alternative way of ending one’s life compared to that provided by physician-assisted suicide (PAS) in the form of ingesting prescribed lethal medication. For example, Mrs. Eddy, an 84-year-old woman suffering from a cascade of debilitating medical conditions, initially sought (illegal) PAS, but then opted to die by VSED [11].

Nevertheless, while VSED is a form of suicide, there remain some important differences between VSED and other forms of suicide such as ingesting an overdose of medication or wrist slashing, which we will call ‘conventional suicide’ (CS). These differences may, to an extent, influence those who erroneously claim that VSED is not suicide. The differences are the following:

1. Refusal to allow VSED for a competent patient involves intervening in the bodily integrity of the person to force feed them against their will, which would be battery at law [12]. Yet, in jurisdictions such as the UK, the law allows the prevention of CS – a police officer or citizen can stop a person from jumping off a bridge [13], as there is a presumption that the person attempting or communicating a plan for suicide is not competent. Individuals attempting or threatening suicide can be committed to a hospital and forced to undergo treatment, provided they are deemed to be incompetent or if relevant mental health legislation applies.
2. Accordingly, there is no right to commit CS. However, competent individuals have a right to undertake VSED, which is grounded in bodily integrity. There is no legal basis for overruling a competent person’s decision to die by VSED, as this would involve forced administration of artificial nutrition and hydration, which a competent person can refuse.
3. Where it is legal, there is still no right to receive PAS, and a physician will not be committing a crime or acting unethically if they refuse the patient’s request even if the patient complies with the legal requirements (the physician may have a right to conscientious refusal or room for judgement that assisted suicide remains inappropriate in a given case). By contrast, the competent patient undertaking VSED has a right to non-interference, and force feeding would be a personal assault [4] (thus, while in PAS a conscientious refusal can take the form of refusing to provide medication to end a competent patient’s life, in VSED it could not take the form of force feeding).

It is possible that the law in some cases has wrongly taken these differences to justify distinguishing VSED from suicide [10]. Furthermore, the typical CS cases may also influence courts’ stance that VSED is not suicide, since there may be a presumption that CS cases connote mental illness and that, in this regard, the VSED cases are not relevantly similar, and thus should not be classified as suicide at all to avoid confusion. Nevertheless, for the reasons stated above, this is not a tenable position. At most, these differences between VSED and CS indicate that there are different types of suicide, with different legal and moral implications, rather than distinguishing VSED *from* suicide.

### Assisting suicide and VSED

Even though we argue that VSED is a form of suicide, we believe it is reasonable to claim that medical practitioners are not assisting suicide when offering standard palliative care treatment for patients undergoing VSED, thereby making VSED more feasible and comfortable for the patient. This view might seem to depend on the wording of the Statutes that make assisting suicide unlawful. ‘Aiding and abetting suicide’ is often used. In Arkansas the phrase ‘assisting in any medical procedure for the express purpose of assisting a patient to intentionally end the patient’s life’ [14] is used – and these words might be defined broadly. Additionally, the words ‘encourage’ and ‘counsel’ are often used, which may include ‘advice’ in some cases, as discussed below.

There is a conceptual difficulty in regarding ‘making a patient comfortable’ as aiding and abetting suicide. If aiding and abetting suicide is a crime, and yet interfering with a competent patient’s decision to undertake VSED is battery, and hence also a crime, then it makes no sense to describe the act of ensuring that a patient is comfortable by virtue of medically indicated palliative care, such as prescribing analgesic drugs to treat pain, as the crime of aiding and abetting suicide. Because there is a right to refuse food and water, palliative care of a patient undertaking VSED cannot be the crime of assisted suicide. Moreover, physicians who provide palliative care for patients undergoing VSED cannot be regarded as agents of these  patients' deaths, unless they do something to accelerate death. However, physicians providing palliative care do not accelerate death with VSED to a greater extent than it is already being accelerated by the patients themselves. Indeed, an essential goal of medicine is to relieve suffering and physicians do no wrong when offering standard medication to relieve the suffering ensued by patients exercising their own autonomous choice [15].

In VSED, physicians are only respecting the competent patient’s autonomous decision, making the patient comfortable, without providing any medical intervention that the patient will use to end their life. At most, physicians wish to help the patient die peacefully, given the patient’s own decision – a decision which is entirely in the patient’s hands. Thus, VSED is different from PAS, where the physician prescribes lethal medication, and thus where the act of causing death is not entirely in the patient’s own hands. In VSED, the doctor simply makes the patient as comfortable as possible when respecting the patient’s autonomous decision to forego food and water. Thus, providing palliative care to patients seeking to die by VSED does not constitute assisted suicide.

Furthermore, in PAS, the physician’s prescription may function, in part, as an authoritative endorsement of the patient’s suicide, possibly detracting from self-determination in ways that cannot be measured by tests for competence or undue influence. In VSED, by contrast, where the physician does not supply the means of effectuating the patient’s choice, there is no such ‘authoritative endorsement’. Finally, in cases where the physician is present when the patient ingests the lethal medication in PAS, there is an element of potential pressure on the patient to go through with it. Thus, VSED is arguably more strongly grounded in self-determination than PAS [4].

There remains a more challenging issue to consider – the provision of advice offering VSED as an option to a patient who may not otherwise have knowledge of it. Consider, in particular, the case where a patient is seeking PAS, but it is illegal, and the doctor mentions the possibility of VSED. The patient replies that they would not choose VSED because it would be too painful, uncomfortable, and distressing both for the patient and their family, yet the physician informs the patient about the possibility of making them comfortable and that other patients have found the process of dying by VSED tolerable (we are indebted to Benjamin White for this example) [15]. Could this be ‘encouraging’ suicide? We believe that the answer is ‘no’, because the physician is advising the patient about exercising a legally protected right. The difference between VSED, which is a right grounded in bodily integrity, and CS, which is not, is paramount here. The same considerations, then, that applied to physician involvement in VSED by virtue of providing palliative care also apply to providing advice about it. Jox et al. [16] argue that VSED is assisted suicide when a physician’s assurance that they can make a patient comfortable is a causally necessary condition of the patient’s undertaking of VSED. However, while the premise about causation may be true, the conclusion that VSED is assisted suicide does not follow. The same causal connection arises in the case of decisions to stop life-prolonging treatment, cases which Jox et al. [16] concede are not suicide. Without palliative care, for instance, a ventilator-dependent patient might decide not to have the ventilation discontinued. Yet, stopping ventilation does not constitute the crime of PAS because of the patient’s right not to be subjected to the burden of ventilation – the same applies to the legal right to refuse food and water.

### Conscientious refusal

While we have argued that patients have a right to undertake VSED and physician involvement in VSED is not assisted suicide, some physicians may regard the practice as morally objectionable since the patient is aiming to end their life. Can physicians ethically refuse to help patients end their lives by means of VSED? The scope and limits of physician refusal to help patients on grounds of conscience is a complex and controversial topic, which cannot be adequately addressed here. We suggest, however, that physicians are not obliged to inform patients seeking to hasten their deaths about the option of VSED, nor are they obliged to provide palliative care for a patient who is determined to undertake it. We believe, however, that, where a patient raises the possibility of VSED themselves, a physician conscientiously opposed to helping a patient undertake VSED should inform the patient that other physicians may be willing to provide palliative care in connection with such.

## Conclusion

The issue of whether physicians, in offering palliative care to patients undertaking VSED, are acting ethically and, in particular, legally, is important because many of those who may be called upon to provide palliative care and help with this option will need to be aware of the legal position. Most authors claim either that VSED is suicide and therefore palliative care of VSED constitutes assisted suicide or, conversely, that VSED is not suicide and thus palliative care of VSED is not assisted suicide. We take a different approach, arguing that VSED is indeed a form of suicide, but that physician provision of palliative care for these patients and even advising on the option, do not constitute assisting suicide. Thus, this conduct would not fall within legislation prohibiting assisted suicide.

## Abbreviations

CS: Conventional suicide
PAS: Physician-assisted suicide
VSED: Voluntarily stopping eating and drinking
